# Comparative analysis of variables that influence behavioral intention to use MOOCs

**DOI:** 10.1371/journal.pone.0262037

**Published:** 2022-04-12

**Authors:** Singha Chaveesuk, Bilal Khalid, Magdalena Bsoul-Kopowska, Eugenia Rostańska, Wornchanok Chaiyasoonthorn

**Affiliations:** 1 KMITL Business School, King Mongkut’s Institute of Technology Ladkrabang, Bangkok, Thailand; 2 Faculty of Management, Czestochowa University of Technology, Czestochowa, Poland; 3 Department of Pedagogy, Faculty of Applied Sciences, WSB University, Warszawa, Poland; The Open University of Hong Kong, HONG KONG

## Abstract

The purpose of this research was to investigate the key factors that influence behavioral intention to adopt MOOCs. The study was conducted in three countries namely, Poland, Thailand, and Pakistan. The study was considered significant considering the advancements in technology that have had an unprecedented impact on education, and the need to conduct learning online due to the COVID-19 to pandemics. The research adopted the Unified Theory of Acceptance and Use of Technology (UTAUT2) and extended it by including other variables including culture, social distancing, and absorptive capacity. The study was conducted using the quantitative methodology, where the data was collected using a structured questionnaire. The data was collected from a sample from each of the three countries, and sample sizes were 455, 490, and 513 for Poland, Thailand, and Pakistan respectively. The data were analyzed using Structural Equation Modeling (SEM) and multi-group SEM analysis. The results of the study indicated that effort expectancy and culture significantly and positively influenced behavioral intention to use MOOCs in all three countries. As well, absorptive capacity is mediated significantly by performance expectancy and effort expectancy. Facilitating conditions have a significant influence on MOOCs in both Thailand and Pakistan. Social influence has a significant influence on behavioral intention to use MOOCs in Thailand, hedonic motivation and price value have a significant influence on behavioral intention to use MOOCs in Poland, and the habit has a significant factor in Pakistan. The keys aspects influencing behavioral intention to Use MOOCs were different in Poland, Thailand, and Pakistan, in various factors which are performance expectancy, social distancing, price value, facilitating conditions, and social influence. The research recommended that it is important to evaluate the situation and prevailing conditions of the concerned country, before implementing the MOOCs and the associated online learning practices.

## 1. Introduction

### 1.1. Massive Open Online Courses (MOOCs)

Advancements in technology have had an unprecedented impact on education. The Massive Open Online Courses (MOOCs) is one of the technologies supporting free learning and education through the internet. MOOCs involve web-based learning programs designed to accommodate large numbers of geographically dispersed students in distance education. MOOCs work by offering an online course to students through the internet [1]. Learning through the internet contains the traditional learning materials that are made accessible online in recorded lectures, course readings, interactive learning modules, online examinations, and online student interaction forums [2, 3]. Initially, MOOCs programs were used as open educational resources (OER) and were not designed to offer academic credits. However, with the advent of the coronavirus pandemic and the need for social distancing, many learning institutions have taken up the idea to offer educational programs in public schools and undergraduate degree programs [4, 5]. The MOOCs have a different approach from the previous online learning programs as it is free and available for anyone to enroll. The development of MOOCs has led to various benefits in learning, including the transformation of higher education for a public good. The educational programs also contribute to the increased opportunity for open connection with global learners.

Educational institutions and universities majorly provide massive Open Online Courses. However, various other entities, including corporate organizations, also offer MOOCs to improve careers and support continuous learning. The term MOOC was first coined in 2008 by the University of Prince Edward about free courses from the institution that promoted connectivity and knowledge sharing. Over the years, MOOCs have developed to include other forms referred to as cMOOCs and xMOOCs [6]. The cMOOCs are massive open online courses that focus on shared interests among an organized online community of learners. The participants’ interests share knowledge in a particular content area. The xMOOCs, on the other hand, are massive Open Online Courses that are centered on tutors. The xMOOCs are centered on the professor and focus on the duplication of knowledge. MOOCs are provided online by various platforms. Some of the common providers of MOOCs include Coursera, Udacity, and edX. The online MOOCs providers collaborate with learning institutions, including universities and colleges, to provide free online courses; however, some charge a small fee for certification purposes.

Inferring from research on the benefits of MOOCs to students [7], the study indicates that the programs offer a variety of courses that students may not be able to enroll in at once due to the tight college schedules. Learning institutions may not offer all the courses that students may be interested in, and this is where the MOOCs can help. Similarly, the MOOCs offer access to learning materials to a diverse range of learners, and this offers an opportunity for students to learn from their peers around the world, especially through the cMOOCs. However, also, there are various challenges for students associated with the use of MOOCs. Gore articulates that courses requiring thorough discussions, the process of discussions can be challenging as the students come from different regions around the world speaking different languages. Also, due to the large number of students in the course classes, the learners can lose attention in learning [8, 9].

During the COVID-19 pandemic, the new education system of MOOCs and its use has become increasingly popular. Many universities have taken their learning programs online, in addition to establishment of many MOOCs platforms. MOOCs are associated with advantages such as flexibility, diversity, free access, and convenience. However, the new education system is associated with high dropout [10]; resistance and fear to the adoption of new technology; and is considered as a disruption to the traditional learning setup. As well, MOOCs requires high level of technology and skills knowledge. Therefore, it is not clear what factors actually could drive towards its adoption, and how each factor influences the behavioral intention to adopt MOOCs. This research, as a result, is geared towards addressing this research gap. This research gap is addressed by two research objectives, (1) to determine the factors influencing the behavioral intention to use MOOCs in Poland, Thailand, and Pakistan, (2) to compare these factors among the three countries.

### 1.2. MOOCs in Thailand, Pakistan, and Poland

The use of MOOCs has increasingly been popular in the past decades since its development in extending online learning to diverse areas around the world. Countries have adopted MOOCs in Asia, Europe, and the USA. Thailand is one of the countries in Asia that has adopted the development of MOOCs to promote digital learning as well as developing digital learning standards to ensure educational quality assurance in Thailand. The MOOC program in Thailand was launched in 2017 through the Thailand Cyber University project (TCU) under the office of the higher education commission. The government sponsors the program through the ministry of education; thus, the courses offered are all free. The country also introduced other forms of MOOCs, including the CHULA MOOC, KMITL Learning Intelligence X, MUx, and SkillLane [11, 12].

In Pakistan, the use of MOOCs to access diverse higher education courses has not been extensively adopted. In 2018, the government of Pakistan conducted a feasibility study through the ministry of education, where the results indicated the need for financial collaboration to ensure a 10% growth in the education sector [13]. Currently, the Allama Iqbal Open University and the Virtual University of Pakistan are the only learning institutions that have adopted the use of MOOCs to offer free online courses in Pakistan. However, the country poses a potential growth in the use of MOOCs in future online learning due to the increasing number of higher learning institutions offering distance education programs. Similarly, the country has a rapidly growing population, with many youths indicating an unprecedented interest in MOOCs provided by international platforms, including Coursera and edX. The programs in Pakistani, however, need to the refined and aligned with the quality-based MOOCs [14].

Poland is the other country in Europe that has increased MOOCs adoption in offering free higher education courses. The development of online learning in Poland has undergone several stages over the years. For instance, MOOC courses were initially provided by stand-alone universities in the country [15]. However, in 2018, there was a huge change in the Polish MOOCs platforms to include a nationwide MOOC platform called Navoica. The name Navoica is about the first Polish university student. The ministry of science developed the Navoica MOOC project in Poland, and higher education offers free educational courses to numerous users in the country [16]. Similarly, over 30 universities and other learning institutions have adopted the Navoica program serving over 30,000 registered users from around Poland [15]. Currently, the Navoica MOOC platform in Poland has managed to offer free online courses to over 12500 users who have received certificates of completion.

Though there is evidence that MOOCs has been adopted in the three countries of Poland, Thailand and Pakistan, no hard research exist on how various factors influences its adoption in each country individually and in comparison. The unexpected disruption of education system by the COVID-19 pandemic forced classes to go online on a short notice. Therefore, MOOCs adoption is a major educational response to the Pandemic. Therefore, evaluating the rate of MOOCs adoption was good, but investigating the factors determining its adoption is vital. Considering the three countries difference in culture and education system, this study compared the findings among themselves.

### 1.3. MOOCs in COVID-19 pandemic period

The COVID-19 pandemic continues to inflict severe consequences on the global economy and other sectors, including the education sector [17–19]. With the shutting down of many economic sectors such as the travel industry, the limitation of movement in many countries led to a massive loss of jobs [20]. Many of the individuals who lost their jobs felt that they would need improvement in skills to get hired in similar positions they held before the COVID-19 pandemic. As such, there was an increased enrollment in online classes through the Massive Open Online Classes (MOOCs). A high number of enrollments for the MOOCs courses during the COVID-19 pandemic was witnessed in provider platforms such as Coursera and Udemy. Among the individuals with high enrollment to the MOOCs programs during the Coronavirus pandemic involved people younger than thirty years and were likely to have advanced degrees. The need for enrollment thus involved advancements in skills [21].

According to Impey and Formanek, the enrollment in MOOCs courses through the Coursera platform during the COVID-19 period skyrocketed to approximately 640% by mid-2020. The increase in enrollment was in part driven by Coursera’s provision of free access to over 3800 courses from the various university partners [22]. Udemy is the other MOOCs provider that saw a significant surge in enrollments during the COVID-19 period. Inferring to [22], there was an applicated 400% surge in enrollment on the Udemy platform for the MOOCs courses in 2020. The increase in the MOOCs enrollments at the platform was driven by the increased lockdowns around the world in the bid to curb the spread of the COVID-19 pandemic. The largest number of enrollments to the MOOCs courses during the COVID-19 pandemic was from the developing countries in Asia, including India and Thailand. However, many of the individuals that enrolled for the MOOCs courses did not complete their courses. The reason for the high number of individuals who did not complete the courses was due to the amount of time for the courses and returned to normal in various regions with the introduction of COVID-19 vaccinations. Many courses under the MOOCs program take up to three years to complete.

Various motivations led to the surge in MOOCs enrollment during the COVID-19 pandemic. Inferring to [23], many individuals enrolled for the online classes through the MOOCs due to the perceived benefits in improving their skills and the chances of getting new better jobs. Also, the increase in MOOCs enrollment during the pandemic period was due to the free nature of the courses offered online. Many of the learners from the developing countries were unable to afford the formal education for the courses in established learning institutions; thus, the MOOCs courses came as an alternative to certification their courses of interest.

## 2. Materials and methods

This study was approved by the Research Ethics Committee of The Management Faculty, Czestochowa University of Technology, Poland with reference number EC-CZE_20_09. The Ethics Committee certified that the study protocol was in compliance with the Declaration of Helsinki, ICH Guidelines for Good clinical practice, and other international guidelines for human research protection. We confirm that the informed consent was obtained voluntarily from all respondents. No information that can identify respondents was included in the questionnaire. They also had the option not to answer any question they feel infringe on their privacy. Participants were assured of confidentiality of any freely provided information in the course of this study with respect to any information that may expose their identity. Consent was given in oral and/or written form.

### 2.1. Theoretical literature

The massive open online courses (MOOCs) present a new trend in higher education learning. The programs present various benefits for the learners, including flexibility in course learning and availability of affordable learning through the free courses. The adoption of the MOOCs programs continues to increase, especially in developing countries, including Thailand, Pakistan, and Poland. However, the key determinant to the success in the adoption of MOOCs is hinged on the students’ levels of acceptance of the learning technologies. Thus, the analysis of the student acceptance in the use of MOOCs can be achieved through the use of the unified theory of acceptance and use of technology (UTAUT2) model [24].

#### 2.1.1. Unified Theory of Acceptance and Use of Technology (UTAUT2)

The MOOCs programs are a new concept in higher education learning and are increasingly becoming significant in the access of education around the world. Understanding the behavior intention to adopt the MOOCs technology programs is thus vital in ensuring the increased adoption among developing countries such as Thailand and Pakistan. The Unified Theory of Acceptance and Use of Technology (UTAUT2) model can be used to analyze the factors influencing MOOCs’ technology acceptance [25–30]. The theory explains the individual’s intentions to use the technologies as well as the subsequent usage behavior. There are various key constructs under the UTAUT2 model that can be used to analyze the behavior intentions of users and the usage behavior of the MOOCs programs.

#### 2.1.2. Behavioral intention to use

The behavioral intention to use involves the motivating factors that influence an individual’s behavior towards particular actions. Under the UTAUT2 theory of behavior, a high likeliness of behavior performance exists where there is a strong intention to perform. The theory considers behavior a being subjective and that individuals are likely to perform particular behaviors where there is the belief that the behavior is approved by the majority [31–33]. The UTAUT2 construct of behavioral intention to use can be applied in the analysis of MOOCs adoption in countries such as Thailand, Poland, and Pakistan. The increased approval of use by peers, friends, and family has a high likelihood of influencing positive behavior towards the use of MOOCs in these countries.

#### 2.1.3. Performance expectancy

The performance expectancy under the UTAUT2 theory involves the level to which individuals perceive a particular technology can help them attain improved performance in their activities. The construct of performance expectancy can be used in the analysis of the adoption of MOOCs in developing countries. With the advancements in technology, the use of e-learning continues to grow as a medium of communication and learning in Pakistan [34]. The growth in the use of MOOCs is thus likely to be viewed as a means to enable the students to achieve their learning interests at a cheaper cost. In understanding the levels of adoption of the MOOCs, there needs to understand the extent of performance expectancy of the learning technologies to the users.

#### 2.1.4. Effort expectancy

The construct of effort expectancy under the UTAUT2 model refers to the degree of belief that the technology under use will be easy to use and effortless. The MOOCs programs are offered through online technological platforms. Thus, understanding the adoption of MOOCs by users, including tutors and students, should be based on the expected ease of use of the technological platforms [35]. Advancements in technology differ in different countries. In developing countries, the ease in use of technology can include the availability of communication technologies and the complexity of use of these technologies and their applications in systems performance [36]. Where there is a high level is the ease of use of technology, there is a likelihood of increased behavioral intention to use the MOOCs programs.

#### 2.1.5. Facilitating conditions

The concept of facilitating conditions is a UTAUT2 construct that links the individual’s behavior actions to the perceptions of the available resources and support for a particular behavior. In the analysis of the key aspects that influence the behavioral intention to use MOOCs, the concept of facilitating faculties explains how the available technical infrastructure can sway the intention to use MOOCs. The MOOCs are provided through online platforms, which include the use of the internet, websites, computers, and supporting learning institution’s infrastructure [37]. The facilitating conditions affect the behavior of the use of the technologies directly. Thus, where there are high levels of facilitating conditions, there will be a great behavioral influence towards the use of MOOCs programs by both students and tutors. However, the facilitating conditions vary from one country to another; and in this case, the facilitating conditions to influence behavior towards the use of MOOCs may include training and other technological applications.

#### 2.1.6. Social influence

Social influence is the other construct of the UTAU2 theory model that links behavioral intention to use with technology’s perceived importance to others. The concept is based on the views and recommendations of others on a particular technology that can influence an individual’s intention to use the technology. Often, the insights of other individuals, including family, friends, and peers, play a vital role in the adoption rates of MOOCs through the belief that they should use the technology, too [38, 39]. A positive social influence on the use of the MOOCs based on the ease of use and the expected benefits plays a positive role in influencing users to use the technologies. Users who believe that important people in their social cycles support their use of MOOCs are more likely to have a high intention of use of the technologies. However, the intentions to use are less to be influenced by individuals who perceive the use of the MOOCs as not supported by among their social networks.

#### 2.1.7. Hedonic motivation

Hedonic motivation is another construct of the UTAUT2 theory that links the behavioral intention to use with the perceived pleasure and enjoyment that is derived from the use of a particular technology. According to Lowry and colleagues [40], technological hedonic motivations include fun, pleasure, and excitement. In their study, the authors articulate that there is a positive influence on behavior towards intentions to use where there is perceived enjoyment of a particular technology. The aspect of hedonic motivation is thus a key determinant in the understanding of the influences of behavioral intention to use MOOCs [41, 42]. For instance, where individuals perceive the use of MOOCs as being easy and enjoyable, there is a positive influence on the intention to use the massive open online courses [43, 44]. However, the intention to use the MOOCs is minimal in individuals who perceive the use of the online technologies supporting the MOOCs as not exciting and enjoyable.

#### 2.1.8. Price value

The other construct under the UTAUT2 theory used to explain the behavioral intention to use includes the price value. Price value refers to the cognitive trade-offs by users between the perceived benefits of using a particular technology and the cost involved in the use of these technologies. The price value under the UTAUT2 theory involves the quality, cost, and price that are likely to influence the user’s intention to use a particular technology [45]. Thus, in the analysis of the adoption of the use of MOOCs, the intention to use would be influenced by the user’s perception of the quality of learning attained through the programs compared to the cost of supporting facilities, including the internet, computers, and cost of the education programs. These factors are important in personal educational decision making and learning intentions especially for the youth due to the importance of learning results in their esteems of the quality of life [46].

#### 2.1.9. Habit

Habit is the other construct of the UTAUT2 theory that can be used to understand the user’s intention to use the massive open online course programs. The concept of Habit is considered under this theory since it is regarded as the degree to which individuals believe behavior to be automatic [47]. Individuals with the Habit of using online learning are likely to develop habitual pressure that significantly influences the user’s intention to use MOOCs. Similarly, the increased Habit of using technology to perform various functions may positively influence the user’s behavioral intention to use MOOCs.

#### 2.1.10. Social distancing

The breakout of the COVID-19 led to various measures aimed at limiting the spread of the virus, including social distancing and lockdowns. With the increasing need for social distancing, the need for various activities, including online learning, increased. The concept of social distancing thus can be used under the UTAUT2 model to explain the possible behavioral intention to use the MOOCs programs users observing social distancing [48]. The interruptions to education through the aspect of social distancing are a major influence on the intention to use MOOCs as many institutions of higher learning shifted to the provision of quality education online. This trend of education development is inevitable due to the growing competition in the educational market [49] which in some cases leads to large-scale redistribution of the intellectual potential [50]. Thus, students requiring quality education while maintaining the social distancing would be positively influenced to adopt the use of MOOCs.

#### 2.1.11. Absorptive capacity

The absorptive capacity is another behavioral aspect that can be integrated with the UTAUT2 model to determine the behavioral intention to use MOOCs. Absorptive capacity involves the ability to value, assimilate and apply new knowledge [39, 51–54]. The absorptive capacity in the adoption of new MOOCs technology involves the user’s capacity to realize the technology’s benefits. Users with high technological efforts have a greater motivation to search for knowledge from other sources. In this case, they are likely to place more value on the external knowledge from the MOOCs programs and, consequently, increase the intention to use the massive open online courses. The absorptive capacity is influenced by the culture of the users [8]. For instance, users who place low value on knowledge are likely to make fewer efforts in using MOOCs to acquire knowledge and consequently will exhibit a low behavioral intention for using the MOOCs technologies.

### 2.2. Empirical literature

Various studies have been conducted in the past to examine the MOOC adoption based on the UTAUT model which was developed from TAM [34, 55]. Inferring from the study by Fianu and Ofori [56] who investigated the factors that affected the use of MOOCs by students in Ghanaian universities, the Unified Theory of Acceptance and Use of Technology (UTAUT) were extended to develop the research model. The results from the research study indicated that constructs that influenced the student’s behavioral intentions to use the facilitating conditions such as computers, including computer self-efficacy, system quality, and performance expectancy. From the research findings, there was limited social influence and effort expectancy that affected the usage of the MOOCs by the students. The researchers thus recommended that the universities should include computer training that could positively influence the facilitating conditions and consequently the MOOC usage intention.

Also, [57] researched the moderating effects of gender on the use of MOOCs through the use of the UTAUT model. In this case, many of the users discontinued their MOOCs programs with an unknown behavioral intention of use. The study thus sought to explain the key factors influencing the behavioral intentions to adopt the MOOCs through the UTAUT model. The research results indicated various constructs of the UTAUT models as the influencing factors, including performance expectancy, social influence, effort expectancy, and self-efficacy. The results from the study thus indicated that there was the minimum effect from gender and experience of the users that influenced their behavioral intentions to use and adopt the MOOCs. The research thus highlighted the factors influencing the behavioral intention to adopt the MOOCs and the limited effects of gender and experience in adopting the technologies.

In the study by [58], the authors conducted an empirical study on MOOC adoption through the application of the UTAUT theory. The purpose of the research involved filling the literature gaps on the factors that promote MOOC adoption in both the teaching and learning context. The study also sought to explain the factors that impeded the successful application of MOOC technologies. The results from the study indicated various UTAUT constructs that positively influenced the adoption of the MOOCs, including performance expectancy as the most influential factor. The performance expectancy factor involved the degree to which individuals perceived the use of the system would help them attain improved performance. The other limiting factor influencing the adoption of the MOOCs from the research included facilitating conditions. Facilitating conditions involve the degree to which individuals believe the existing technological infrastructure could support the system.

The adoption of MOOCs as practiced by both the students and the tutors was also investigated. [59] investigated teacher’s adoption of MOOCs using the UTAUT2 theory. According to the study, despite the increase in popularity in the use of MOOCs globally, there has been little attention from tutors. Tutors find it challenging to use MOOCs, thus affecting the rate of adoption by users around the globe. From the research, there are various constructs under the UTAUT model that affect the rate of adoption of MOOCs by tutors. For instance, aspects of social influence, the facilitating conditions, price value, and performance expectancy played a significant role in influencing the rate of tutor adoption of the MOOCs. However, from the research, other constructs failed to influence the teacher’s adoption of the MOOCs, including hedonic motivation and effort expectancy.

The Unified Theory of Acceptance and Use of Technology (UTAUT) uses various constructs to explain the behavioral intentions to use. In the research by [24], the researchers used the perceived value construct of the UTAUT theory to understand the factors influencing the behavioral intention to use MOOCs. The purpose of the research also included the validation of the use of the UTAUT model in the adoption of MOOCs. From the research findings, the perceived value significantly influenced the intention to use MOOCs among many users. The perceived value involves the trade-off between the benefits of the technology under a user with the cost of using the technologies. The other UTAUT theory constructs in the research that influenced the adoption of the MOOCs involved performance expectancy, effort expectancy, and social influence.

The behavioral intentions to use are influenced by various factors that can be explained through the UTAUT theory. The behavior can be witnessed where the use of technology is involved. [60] sought to investigate the intention to adopt mobile payments using the UTAUT theory. The phenomenon of using mobile is growing around the world, and it offers users increased flexibility and convenience to perform their daily activities with the use of mobile payments. An analysis using the UTAUT theory on the Brazilian users on the adoption of the mobile payments indicated that various constructs of the UTAUT theory on behavioral intention had a significant role in influencing the adoption got mobile payment technologies. However, the construct of perceived cost had limited influence on the users’ behaviors on intentions to use.

Also, the research study by [61] investigates the consumer acceptance and use of information technology using the UTAUT analysis. The research hypothesized various aspects of users to influence the behavioral intention to use information technology, such as gender, age, and experience. The research also analyzed the data based on three constructs of the UTAUT2 model, including hedonic motivation, price value, and Habit. Based on the unified theory of acceptance and use of technology theory, hedonic motivation involves the enjoyment derived from the use of technology, while price value involves a trade-off between the perceived benefits from the adoption of the technology and the cost of using the technologies. Also, the habit construct involves the level with which the users tend to perform behavior automatically. From the research, results indicated that UTAUT constructs of Habit and hedonic motivation played a significant role in the adoption and acceptance of information technology.

In the study by [62], the UTAUT2 model was used to analyze the public acceptance level of automated cars in Europe. With the advancements in technology, the development of conditionally automated cars needed public acceptance to be adopted for use. Using the UTAUT2 theory to investigate the intention to use behavior by the public revealed various constructs influencing behavior. For instance, the research applied the analysis of constructs including social influence, facilitating conditions, performance and effort expectancy, and hedonic motivation. Results from the structural analysis in the research indicated that the behavioral intention to use the conditioned automated cars by the public in select European countries was influenced by UTAUT2 constructs, including hedonic motivation, social influence, and performance expectancy.

### 2.3. Conceptual framework

**Fig 1:**
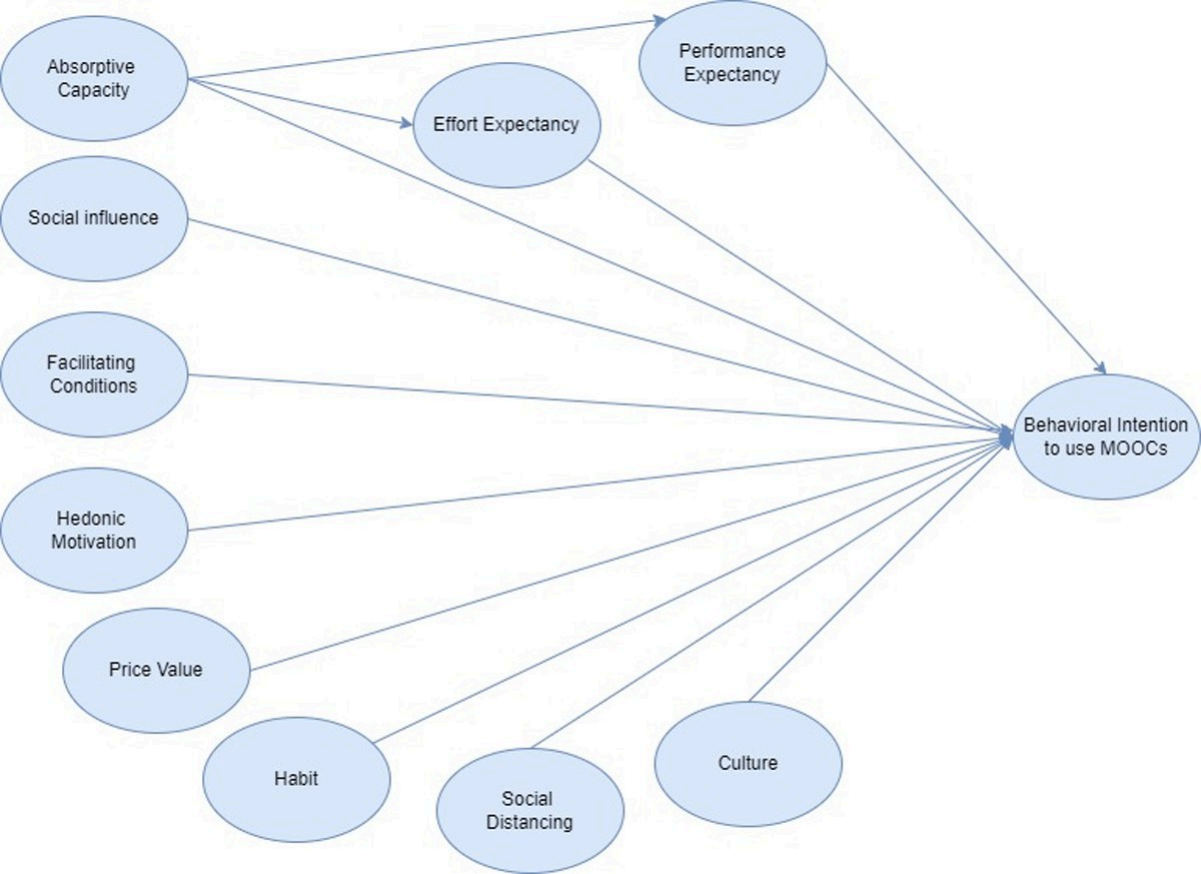
Conceptual framework.

From the evaluation of the literature review and the adopted theoretical model, the conceptual framework was developed. The conceptual framework has 9 independent variables and one dependent variable. The independent variables include performance expectancy (PE), and effort expectancy (EE), absorptive capacity (AC), social influence (SI), facilitating conditions (FC), hedonic motivation (HM), price value (PV), habit (HB), social distancing (SD), culture (CL). The dependent variable was the behavioral intention to use (BI). The proposed conceptual framework is presented in the Fig 1 below.

From the above conceptual framework, the following hypothesis was developed:

**H1:** Performance expectancy has a positive effect on behavioral intention to use MOOCs
**H2:** Effort expectancy has a positive effect on behavioral intention to use MOOCs
**H3:** Absorptive capacity has a positive effect on behavioral intention to use MOOCs
**H4:** Social Influence has a positive effect on behavioral intention to use MOOCs
**H5:** Hedonic motivation has a positive effect on behavioral intention to use MOOCs
**H6:** Facilitating conditions has a positive effect on behavioral intention to use MOOCs
**H7:** Price Value has a positive effect on behavioral intention to use MOOCs
**H8:** Social distancing has a positive effect on behavioral intention to use MOOCs
**H9:** Culture has a positive effect on behavioral intention to use MOOCs
**H10** Habit has a positive effect on behavioral intention to use MOOCs
**H11:** The effect of absorptive capacity on behavioral intention to use MOOCs is mediated by performance expectancy and effort expectancy
**H12:** The important variables influencing behavioral intention to Use MOOCs are similar in Poland, Thailand, and Pakistan

Based on the above model and literature review, the measurement scales were developed as presented in the Table 1 below. The scales were adjusted to the questions for the current study.

**Table 1:** Measurement scales.

Latent Variables	Scales	Sources
Behavioral Intention to Use	I intend to use MOOCs immediately	[38, 39, 63]
	I will use MOOCs in future learning sessions	
	I will recommend others to use MOOCs	
Performance Expectancy	Using MOOCs enables me to accomplish my learning activities more quickly	[38, 39, 63]
	Using MOOCs improves my learning performance (e.g., develop new skills, techniques and gain experience)	
	Using MOOCs enables me to learn more quickly as compared to traditional classroom	
Effort Expectancy	Learning to operate MOOCs would be easy for me	[38, 39, 63]
	My interaction with MOOCs would be clear and understandable	
	I find MOOCs to be flexible to interact with	
	I believe I require little effort to understand how MOOCs works	
Social Influence	People who influence my behavior think that I should use MOOCs	[38, 39, 63]
	People who are important to me think I should use MOOCs	
	People who use MOOCs enjoy more prestige than those who do not	
	People who use MOOCs have a status symbol in my environment	
Facilitating Conditions	I have necessary resources to use MOOCs	[38, 39, 63]
	I have necessary knowledge to use MOOCs	
	Guidance is available to me in the selection of MOOCs	
	Specialized instructions concerning the MOOCs was available to me	
Absorptive Capacity	I am able to acquire information using MOOCs for my learning activities	[38, 39, 63]
	I am able to learn through interactive discussions forum using MOOCs	
	I am able to share important knowledge using MOOCs	
Price Value	Learning through MOOCs is worth more than the time and effort given to it	[63, 65]
	MOOCs given me the opportunity to decide about the pace of my own learning	
	MOOCs gives me the opportunity to increase my knowledge and to control my success (e.g., quizzes, assignment, assessments, etc.)	
Hedonic Motivation	Using MOOCs is fun	[63, 65]
	I enjoy using MOOCs	
	Using MOOCs is very entertaining	
Habit	The use of MOOCs has become habit for me	[63, 65]
	I am addicted to using MOOCs to accomplish my study tasks	
	Using MOOCs has become natural for me	
Social Distancing	Using MOOCs will help me reduce the chances of getting infected with COVID-19	[5, 66, 67]
	I feel confident in my ability to engage in social distancing	
	Using MOOCs enables good interaction with the other students enrolled	
Culture	I get better learning results when I study as a MOOC group member that when I study independently on my own	[9, 64]
	Studying MOOCs, rules, and regulations are important because they inform me what is expected of me	
	It is important to have detailed learning outcomes in details so that I always know what I’m expected to study	

### 2.4. Methodology

This research on “variables that influence behavioral intention to Use MOOCs–Comparing Poland, Thailand, and Pakistan” aimed to investigate the factors that influence behavioral intention to use MOOCs in these three countries and compare their results. The study adopted the UTAUT2 model but incorporated additional variables of social distancing and culture. The social distancing aspect was considered vital considering the influence of COVID-19 on the education system with social distancing as one of the measures of preventing contracting the virus. Education has culturally occurred in face-to-face environment, the significance of culture here is to ascertain the acceptance and adoption of a cultural shift that sees education and learning occurring in online environments either synchronously or asynchronously; therefore, the study intended to determine how social distancing influences the intention to adopt MOOCs. The culture was considered important due to the changing nature of learning, and the varying cultural background in the three countries. Therefore, it was important to evaluate whether culture was a contributing factor towards the adoption of MOOCs. Mixed research methodology was applied to investigate the factors influencing the intention to use MOOCs incorporating quantitative research technique. Data was collected from the study population using a structured questionnaire and the collected data were analyzed using statistical software (Amos v26) to answer the research questions and evaluate the research hypotheses.

The study populations were professionals and students who have used or were using the MOOCs in their studies in Poland, Thailand, and Pakistan. The population also included teachers and lecturers who were delivering content and teaching courses online through MOOCs. Since this population is large, representative samples in each country were selected. The stratified sampling design was adopted to develop samples from the population, but the specific respondents were chosen randomly. The selected sample sizes were 455, 490, and 513 for Poland, Thailand, and Pakistan respectively. During the entire research, the ethics process observed confirmed to the set standards. All the laid down protocols was observed, including informed consent of the institution and respondents, guarantee of their anonymity and free withdrawal.

The questionnaire was structured using a five-point Likert scale where 1 = strongly disagree and 5 = strongly agree. For each of the latent variables, several questions were used to collect the data. The questions for each variable were developed with references from previous studies [4, 13, 22, 32, 38, 39, 58, 68]. The data analysis was conducted using several techniques. First, descriptive statistics were conducted to evaluate the demographic characteristics of the respondents. The second was testing and evaluation of the model, to ascertain its suitability, validity, and reliability. This was conducted using CFA, Cronbach’s alpha, and convergent reliability. The structural equation modeling (SEM) was used to evaluate the hypothesis of the study, while the multi-group SEM analysis was used to compare the results of the three countries [69].

## 3. Results

### 3.1. Demographic characteristics

This section evaluated the demographic characteristics of the respondents. The first variable was gender, whereas, in Poland, males were the majority (72.5%), in Thailand and Pakistan; females were the majority 59% and 71.5% respectively. Considering age, the majority age group for all the countries were 26–35 years, represented by 69.7%, 71.8%, and 67.6% for Poland, Thailand, and Pakistan. The second-largest age group was 18–25 years, for all the countries with 15.4%, 20.8%, 18.9% for Poland, Thailand, and Pakistan respectively. Another variable evaluated was education level where the largest education level for all the countries was bachelor’s degree with 58.5%, 87.3%, and 59.5% for Poland, Thailand, and Pakistan respectively. The computer knowledge of the respondents indicated that the majority of respondents in Poland indicated Good computer knowledge (40.4%), moderate computer knowledge (58%) for Thailand, and Good (40.5%) for Pakistan. For the internet knowledge majority of Poland respondents indicated they have good (45.9%) internet knowledge, in Thailand, they indicated moderate (44.1%), and in Pakistan indicated good (46.8%) internet knowledge. Another variable that was evaluated was internet consumer, where the majority in Poland indicated that have used internet for more than 10 years (35.4%), similar to Thailand (54.5%), but for Pakistan it was majority have used internet between 6–10 years (34.5%). For the internet usage variable, the majority of respondents in Poland indicated they use the internet for more than 3 hours daily (61.3%), for Thailand the majority is 2–3 hours daily (88%) and for Pakistan, it is more than three hours (61%). A summary of the demographic information is presented in Table 2.

**Table 2:** Descriptive statistics of the demographics.

		Poland		Thailand		Pakistan	
		n	%	n	%	n	%
**Gen**							
	Male	330	72.5	201	41	367	71.5
	Female	125	27.5	289	59	146	28.5
**Age**							
	18–25 Years	70	15.4	102	20.8	97	18.9
	26–35 Years	317	69.7	352	71.8	347	67.6
	36–45 Years	52	11.4	26	5.3	52	10.1
	46–55 Years	10	2.2	9	1.8	10	1.9
	55 and above	6	1.3	1	0.2	4	1.4
**Education**							
**Level**	Junior High School or Lower	2	0.4	2	0.4	2	0.4
	High School / Diploma	10	2.2	9	1.8	11	2.1
	Bachelor’s Degree	266	58.5	428	87.3	305	59.5
	Post-Graduate or Higher	177	38.9	51	10.4	195	38
**Computer**							
**Knowledge**	Very Poor	8	1.8	37	7.6	9	1.8
	Poor	33	7.3	115	23.5	34	6.6
	Moderate	157	34.5	284	58	183	35.7
	Good	184	40.4	47	9.6	208	40.5
	Very Good	73	16	7	1.4	79	15.4
**Internet**							
**Knowledge**	Very Poor	5	1.1	80	16.3	6	1.2
	Poor	22	4.8	180	36.7	24	4.7
	Moderate	120	26.4	216	44.1	138	26.9
	**Good**	209	45.9	14	2.9	240	46.8
	Very Good	99	21.8			105	20.5
**Internet**							
**Consumer**	Don’t Use	4	0.9			4	0.8
	1–5 Years	135	29.7	26	5.3	161	31.4
	6–10 Years	155	34.1	197	40.2	177	34.5
	More than 10 Years	161	35.4	267	54.5	171	33.3
**Internet**							
**Usage**	Less than 1 Hour	15	3.3	10	2	17	3.3
	1–2 Hours	66	14.5	49	10	74	14.4
	2–3 Hours	95	20.9	431	88	109	21.2
	More than 3 Hours	279	61.3			313	61

### 3.2. Evaluation of the model

The first evaluation of the model was confirmed using Confirmatory Factor Analysis (CFA) test. CFA was used to evaluate how well the measured variables represented the number of the construct. The CFA for each country was conducted independently. The first CFA was for Poland, then Thailand, and lastly Pakistan. The first CFA model revealed unsatisfactory results. But after improving the model by developing error covariance as suggested by modification indices, and removal of the paths with less than 7.0 standardized regression weights, the model improved and met the required thresholds, as summarized in Table 3 below. To improve the model, some observant variables were deleted including AC1, PV2, HB1, HB2, SD4, and CL1. It is critical to note that since the purpose of this research is to perform multi-group analysis, the CFI model was moderated in a way it fit and improved a single model to use in multi-group SEM analysis. After getting satisfied with CFA, the model was considered suitable for conducting the SEM analysis.

**Table 3:** Model fitness evaluation statistics.

		Poland		Thailand		Pakistan	
Model Fit Index	Threshold	First Model	Improved Model	First Model	Improved Model	First Model	Improved Model
**X** ^**2**^ **/df**	Value of <2.0 (Hu & Bentler, 1999, and <5.0 (Wheaton et al, 1977)	2.623	2.085	2.723	2.166	3.623	2.448
**RMSEA**	Value between .08 to .10 (mediocre fit), < .08 (goof fit) (MacCallum et al., 1996)	0.060	0.049	0.078	0.049	0.088	0.053
**TLI**	Value of ≥.90 (Bentler, 1990) and ≥.95 (Hu & Bentler, 1999)	0.892	0.938	0.890	0.933	0.874	0.930
**IFI**	Value of >.90 Bentler and Bonnet (1980)	0.904	0.947	0.901	0.943	0.921	0.941
**CFI**	Walue of ≥.90 (Bentler, 1990)	0.903	0.947	0.899	0.943	0.872	0.940
**NFI**	Value >.90 or >.95 (Miles & Shevlin, 1998)	0.853	0.904	0.872	0.900	0.885	0.904
**GFI**	Value of ≥.90 (Bentler, 1990); >0.8 acceptable (Baumgartner and Homburg, 1995)	0.836	0.877	0.812	0.877	0.832	0.876

### 3.3. Reliability and validity analysis

The reliability and validity of the model were evaluated as well. The reliability was evaluated using composite reliability (CR) while validity was evaluated using convergent validity measured using average variance extracted (AVE). According to the threshold proposed by [70, 71], AVE should be greater than 5.0 while CR should be greater than 7.0. From the results presented in Table 4, the two thresholds were met, confirming the suitability of the model’s validity and reliability. This confirmation led to the next step of evaluating the hypotheses of the study using the SEM analysis.

**Table 4:** Reliability and validity analysis.

	Poland		Thailand		Pakistan	
	CR	AVE	CR	AVE	CR	AVE
**AC**	0.856	0.665	0.821	0.605	0.854	0.662
**CL**	0.837	0.631	0.803	0.576	0.840	0.637
**BI**	0.850	0.587	0.841	0.569	0.861	0.609
**SD**	0.793	0.562	0.774	0.533	0.808	0.585
**HB**	0.831	0.562	0.805	0.538	0.713	0.543
**FC**	0.842	0.572	0.835	0.559	0.841	0.571
**SI**	0.848	0.582	0.821	0.535	0.855	0.596
**HM**	0.858	0.602	0.834	0.557	0.883	0.653
**PE**	0.842	0.572	0.842	0.571	0.850	0.587
**EE**	0.854	0.594	0.853	0.592	0.882	0.653
**PV**	0.729	0.559	0.780	0.510	0.722	0.651

Performance expectancy (PI), Effort expectancy (EE), Absorptive capacity (AC), Social Influence (SI), Hedonic motivation (HM), Facilitating conditions (FC), Price Value (PV), Social distancing (SD), Culture (CL), Habit (HB), Behavioral intention to use MOOC (BI).

### 3.4. Hypothesis evaluation

After being satisfied with the proposed model, the hypothesis of the study was evaluated by conducting Structural Equation Modeling (SEM). Hypotheses 1 to hypothesis 10 were evaluated using SEM for each country individually, while hypothesis 11 was evaluated by conducting a multi-group SEM analysis to compare results in the three countries. The results are discussed in the following section.

#### 3.4.1. SEM analysis for Poland

**Fig 2:**
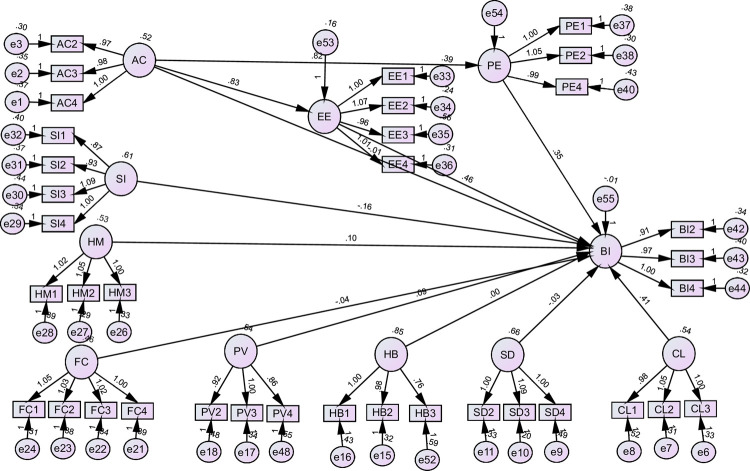
SEM analysis for Poland.

Fig 2 illustrates the SEM analysis revealed that the path coefficient between PE and BI **(HI)** was β = 0.352, p<0.01, hence the hypothesis was supported that Performance expectancy has a positive effect on behavioral intention to use MOOCs in Poland. The path coefficient between EE and BI **(H2)** was β = 0.457, p<0.01 which supported hypothesis 2 that effort expectancy has a positive effect on behavioral intention to use MOOCs. The path coefficient between AC and BI **(H3)** revealed that β = -0.014, p>0.01 which led to the rejection of hypothesis 3 that ‘absorptive capacity has a positive effect on behavioral intention to use MOOCs’. The path coefficients between SI and BI **(H4)** indicated that β = -163, p<0.01 which led to the rejection of hypothesis 4 that “social Influence has a positive effect on behavioral intention to use MOOCs”. The fifth hypothesis **(H5)** was represented by the path coefficients between HM and BI (β = 0.100, p<0.01) which confirmed the hypothesis. Hypothesis six **(H6)** was not supported because the path between FC and BI was not significant. The path coefficient between PV and BI **(H7)** indicated that β = 0.088, p<0.01 which supported the hypothesis that price value has a positive effect on behavioral intention to use MOOCs. The path coefficient between SD and BI **(H8)** indicated that β = -0.030, p> 0.05 which failed to confirm the hypothesis. The path coefficients between CL and BI **(H9)** indicated that β = 0.413, p<0.01 which confirmed hypothesis 9 that "culture has a positive effect on behavioral intention to use MOOCs". The path coefficient between HB and BI **(H10**) indicated that β = 0.003, p>0.01 which led to the rejection of hypothesis 10. The results of the tested hypotheses have been summarized in Table 5.

**Table 5:** SEM analysis for Poland.

Hypothesis	Paths			β	Supported?
**H1**	PE	---->	BI	.352***	Yes
**H2**	EE	---->	BI	.457***	Yes
**H3**	AC	---->	BI	-.014	No
**H4**	SI	---->	BI	-.163***	No
**H5**	HM	---->	BI	.100***	Yes
**H6**	FC	---->	BI	-.043	No
**H7**	PV	---->	BI	.088***	Yes
**H8**	SD	---->	BI	-.030	No
**H9**	CL	---->	BI	.413***	Yes
**H10**	HB	---->	BI	.003	No
**H11**	AC----> PE----> BI			.289**	Yes
	AC----> EE----> BI			.381**	Yes

*** significant at 0.01

** significant at 0.05; Performance expectancy (PI), Effort expectancy (EE), Absorptive capacity (AC), Social Influence (SI), Hedonic motivation (HM), Facilitating conditions (FC), Price Value (PV), Social distancing (SD), Culture (CL), Habit (HB), Behavioral intention to use MOOC (BI).

#### 3.4.2. SEM analysis for Thailand

**Fig 3:**
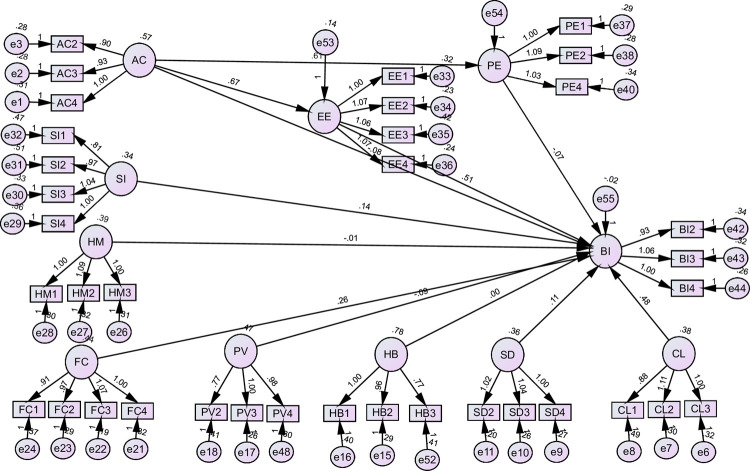
SEM analysis for Thailand.

Fig 3 and Table 6 evaluated the hypothesis for the case of Thailand. The results of the data indicated that among the variables evaluated, several hypotheses were supported. The supported hypothesis were the path coefficient between EE and BI for hypothesis 2 (**H2**) (β = 0.511, p < 0.01); path coefficient between SI and BI for hypothesis 4 (**H4**) (β = 0.143, p < 0.01); the path coefficients between FC and BI for hypothesis 6 (**H6**) (β = 0.262, p < 0.01); path coefficients between SD and BI for hypothesis 8 **(H8)** (β = 0.110, p < 0.01); and path coefficients between SD and BI for hypothesis 9 **(H9)** (β = 0.475, p < 0.01). These hypotheses were supported because independent variables significantly influenced (p-value < 0.05) the dependent variable. However, the insignificant path coefficients that did not support the hypothesis included PE and BI **(H1);** AC and BI **(H3);** HM and BI **(H5);** PV and BI **(H7);** and HB and BI **(H10)**.

**Table 6:** SEM analysis for Thailand.

Hypothesis	Paths			β	Supported
**H1**	PE	---->	BI	-.075	No
**H2**	EE	---->	BI	.511***	Yes
**H3**	AC	---->	BI	-.082	No
**H4**	SI	---->	BI	.143***	Yes
**H5**	HM	---->	BI	-.013	No
**H6**	FC	---->	BI	.262***	Yes
**H7**	PV	---->	BI	-.091***	No
**H8**	SD	---->	BI	.110***	Yes
**H9**	CL	---->	BI	.475***	Yes
**H10**	HB	---->	BI	-.004	No
**H11**	AC----> PE----> BI			-.046**	Yes
	AC----> PE----> BI			.340**	

*** significant at 0.01

** significant at 0.05; Performance expectancy (PI), Effort expectancy (EE), Absorptive capacity (AC), Social Influence (SI), Hedonic motivation (HM), Facilitating conditions (FC), Price Value (PV), Social distancing (SD), Culture (CL), Habit (HB), Behavioral intention to use MOOC (BI).

#### 3.4.3. SEM analysis for Pakistan

**Fig 4:**
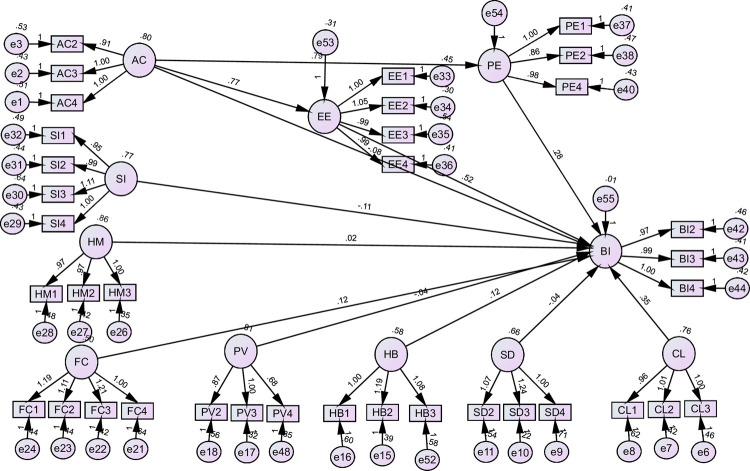
SEM analysis for Pakistan.

This section evaluated the hypothesis for the case of Pakistan. Among the hypothesis that were supported by the statistics include: the path coefficient between PE and BI for Hypothesis 1 **(H1)** (β = 0.276, p < 0.01); path coefficient between EE and BI for Hypothesis 1 **(H2)** (β = 0.517, p < 0.01); path coefficient between FC and BI for Hypothesis 1 **(H6)** (β = 0.121, p < 0.01); path coefficient between CL and BI for Hypothesis 1 **(H9)** (β = 0.355, p < 0.01); and path coefficient between HB and BI for Hypothesis 1 **(H10)** (β = 0.123, p < 0.01). The path coefficients for these relationships were positive and statistically significant (p-value < 0.05) and therefore, the associated hypothesis was supported. However, the path coefficients that were not statistically significant included the path coefficients of the relationships between AC and BI **(H3)**; AC and BI **(H3)**; SI and BI **(H4)**; HM and BI **(H5)**; PV and BI **(H7)**; and SD and BI **(H8)**. Table 7 and Fig 4 presents the SEM analysis for Pakistan.

**Table 7:** SEM analysis for Pakistan.

Hypothesis	Paths			β	Supported
**H1**	PE	---->	BI	.276***	Yes
**H2**	EE	---->	BI	.517****	Yes
**H3**	AC	---->	BI	-.082	No
**H4**	SI	---->	BI	-.114***	No
**H5**	HM	---->	BI	.025	No
**H6**	FC	---->	BI	.121***	Yes
**H7**	PV	---->	BI	-.045	No
**H8**	SD	---->	BI	-.042	No
**H9**	CL	---->	BI	.355***	Yes
**H10**	HB	---->	BI	.123***	Yes
**H11**	AC----> PE----> BI			.219**	Yes
	AC----> EE----> BI			.399**	Yes

*** significant at 0.01

** significant at 0.05; Performance expectancy (PI), Effort expectancy (EE), Absorptive capacity (AC), Social Influence (SI), Hedonic motivation (HM), Facilitating conditions (FC), Price Value (PV), Social distancing (SD), Culture (CL), Habit (HB), Behavioral intention to use MOOC (BI).

#### 3.4.4. Multi-group SEM analysis

The purpose of this analysis was to compare the three countries (Poland, Thailand, and Pakistan) in terms of important variables that influence behavioral intention to use MOOCs. This was addressed in the last hypothesis (H12) of the study. The moderating variable for the analysis was country (Poland = 1; Thailand = 2, Pakistan = 3). The Chi-square differential technique was adopted to compare the difference between the three groups. Before the chi-square analysis, the SEM results for three countries were compared as presented in Table 8.

**Table 8:** Multi-group SEM analysis.

			Poland		Thailand		Pakistan	
			β	p-value	β	p-value	β	p-value
AC	---->	PE	0.821	***	0.611	***	0.791	***
AC	---->	EE	0.834	***	0.666	***	0.772	***
PE	---->	BI	0.352	***	-0.075	0.151	0.276	***
EE	---->	BI	0.457	***	0.511	***	0.517	***
CL	---->	BI	0.413	***	0.475	***	0.355	***
SD	---->	BI	-0.03	0.237	0.11	***	-0.042	0.11
HB	---->	BI	0.003	0.883	-0.004	0.835	0.123	***
PV	---->	BI	0.088	0.003	-0.091	***	-0.045	0.071
HM	---->	BI	0.1	***	-0.013	0.649	0.025	0.291
FC	---->	BI	-0.043	0.162	0.262	***	0.121	***
SI	---->	BI	-0.163	***	0.143	***	-0.114	***
AC	---->	BI	-0.014	0.899	-0.082	0.198	-0.082	0.261

*** significant at 0.01

** significant at 0.05; Performance expectancy (PI), Effort expectancy (EE), Absorptive capacity (AC), Social Influence (SI), Hedonic motivation (HM), Facilitating conditions (FC), Price Value (PV), Social distancing (SD), Culture (CL), Habit (HB), Behavioral intention to use MOOC (BI).

To conduct the chi-square test, the unconstrained and fully constrained models were developed. To obtain the unconstrained model, the insignificant paths were trimmed for all the countries, starting with the one with the least insignificant paths. In this case, paths AC----> BI for all countries, HM----> BI for Thailand and Pakistan, and HB----> BI for Poland and Thailand were constrained to obtain the fully constrained model. The fully constrained model was obtained by setting the regression weights to equal to each other. The unconstrained and fully constrained models, chi-square, and degrees of freedom (df) are recorded in Table 9 below.

**Table 9:** Chi-square test results.

* *	Chi-square	d.f.	p-value	Invariant?
Overall Model				
Unconstrained	13994.434	1755		
Fully constrained	14154.928	1823		
Number of groups		3		
Difference	160.494	68	0.000	NO
**Chi-square Thresholds**				
*95% Confidence*	14000.43	1757		
Difference	5.99	2	0.050	
*99% Confidence*	14003.64	1757		
Difference	9.21	2	0.010	

From the results presented above, the chi-square difference was 160.494, df = 68 and p-value = 0.000. Since the p-value (0.000) < 0.05 and 0.01 at 95% and 99% confidence level, the three countries were not invariant. In other words, hypothesis 12 **(H12)** was rejected, which implied that keys aspects influencing behavioral intention to Use MOOCs different in Poland, Thailand, and Pakistan. Since the overall model was not invariant, it was important to conduct path-by-path analysis. The chi-square threshold for path-by-path analysis was 14000.43 (95% confidence level) and 14003.64 (99% confidence level) as presented in Table 10. From the results, it was found that important variables influencing behavioral intention to use MOOCs were different among the three countries in several variables, which are performance expectancy (PE), social distancing (SD), price value (PV), facilitating conditions (FC) and social influence (SI).

**Table 10:** Path by path analysis.

Threshold	Paths			Path Chi-square	Variant?
14000.43 (95% CL)	PE	---->	BI	14027.304	Yes
	EE	---->	BI	13995.080	No
	CL	---->	BI	13996.477	No
14003.64 (99% CL)	SD	---->	BI	14001.297	Yes (95%)
	PV	---->	BI	14003.603	Yes (95%)
	FC	---->	BI	14008.358	Yes
	SI	---->	BI	14016.440	Yes

## 4. Discussions

This section evaluated the findings of the study, about the previous research findings. Firstly, a summary of the findings for all the countries was conducted as presented in Table 11 below.

**Table 11:** Summary of hypothesis results.

Hypothesis	Paths			β	Supported?	β	Supported?	β	Supported?
				Poland		Thailand		Pakistan	
**H1**	PE	---->	BI	.352***	Yes	-.075	No	.276***	Yes
**H2**	EE	---->	BI	.457***	Yes	.511***	Yes	.517****	Yes
**H3**	AC	---->	BI	-.014	No	-.082	No	-.082	No
**H4**	SI	---->	BI	-.163***	No	.143***	Yes	-.114***	No
**H5**	HM	---->	BI	.100***	Yes	-.013	No	.025	No
**H6**	FC	---->	BI	-.043	No	.262***	Yes	.121***	Yes
**H7**	PV	---->	BI	.088***	Yes	-.091***	No	-.045	No
**H8**	SD	---->	BI	-.030	No	.110***	Yes	-.042	No
**H9**	CL	---->	BI	.413***	Yes	.475***	Yes	.355***	Yes
**H10**	HB	---->	BI	.003	No	-.004	No	.123***	Yes
**H11**	AC----> PE----> BI			.289**	Yes	-.046**	Yes	.219**	Yes
	AC----> EE----> BI			.381**		.340**		.399**	
**H12**	p-value = 0.000				No				

*** significant at 0.01

** significant at 0.05; Performance expectancy (PI), Effort expectancy (EE), Absorptive capacity (AC), Social Influence (SI), Hedonic motivation (HM), Facilitating conditions (FC), Price Value (PV), Social distancing (SD), Culture (CL), Habit (HB), Behavioral intention to use MOOC (BI).

This research considered it important to discuss the findings based on the three countries. The statistics showed that effort expectancy has a significant and positive effect on behavioral intention to use MOOCs for all the three countries of Poland, Thailand, and Pakistan. These findings are supported by [68, 72–75] who investigated the factors influencing the use of mobile eCommerce. Their results indicated that effort expectancy has a significant influence on the behavioral intention to use mobile commerce technology. Similar results were obtained by [75] who investigated the factors affecting the user acceptance of the mobile internet using the UTAUT model. Among the factors that were found to have a significant influence on mobile internet usage was effort expectancy. These finds portray a picture that the individuals’ expectations regarding the effort needed in using MOOCs is a critical factor in determining the MOOCs adoption intention. In these cases, the aspects that are considered important include the ease of use of the technology, clarity and ability to understand by the users, flexibility to interact in the platform as well as the effort required to understand how the whole MOOCs system works. These are the factors that the facilitators should consider when implementing MOOCs.

Another variable that was found to have a significant and positive effect on behavioral intention to use MOOCs was culture. From the findings of the study, the culture among the three countries of Poland, Thailand, and Pakistan is different, and hence the variation significantly influenced their MOOCs use. From the research, a one-unit increase in cultural aspects would result in 0.413, 0.475, and 0.355 increase in behavioral intention to use MOOCs in Poland, Thailand, and Pakistan respectively. These findings are supported by that of [9] who investigated the cross-cultural approach towards the adoption of open educational resources in higher education. The findings indicated that culture was among the variables that have the strongest influence on behavioral intention to use open education resources. Another hypothesis that was accepted in all countries was hypothesis 11, that the effect of absorptive capacity on behavioral intention to use MOOCs is mediated by performance expectancy and effort expectancy. Though absorptive capacity was found to have no significant effect on behavioral intention to use MOOCs in all three countries, it was mediated to a significant influencer by performance expectancy and effort expectancy. These finding bring in a critical aspect that as far as adoption of MOOCs is concerned, the cultural aspects of the concerned students should be put into considerations. The cultural aspects that should be put into considerations include the rules, policies, beliefs, and norms that are observed by the concerned individuals.

Another important consideration was the variables that have a significant effect on MOOCs in two countries. For instance, for Thailand and Pakistan, the facilitating condition was found to have a significant effect on behavioral intention to use MOOCs. A one-unit increase in facilitating conditions would lead to a 0.262 and 0.121 units increase in behavioral intention to use MOOCs. These findings are in line with those of [75] who researched the factors that influence the users’ behavioral intention to use the online booking system, for car services as the Car Service Centre in Malacca. Their results indicated that facilitating conditions are among the factors that positively influence behavioral intention to use online booking systems. From the findings of this study, it is argued that for Thailand, and Pakistan, the extent to which the existing organization, infrastructural facility, and technical aspects determines how the MOOCs technology will be adopted. Therefore, the aspects such as internet availability, computers, tablets and laptop devices to use are critical facilitating factors that determine the adoption of MOOCs.

All the remaining variables have a significant effect on behavioral intention to use MOOCs in only one country. For instance, while social influence was found to have a significant influence on behavioral intention to use MOOCs in Thailand, hedonic motivation and price value were found to have a significant influence on behavioral intention to use MOOCs in Poland. Additionally, the habit was found to be a significant factor in Pakistan as far as behavioral intention to use MOOCs is concerned. This could be attributed to the strong sense of cultural and social aspects experienced and present in Pakistan. Regarding the social influence, the MOOCs facilitators in Thailand and should consider it an important aspect. This could be attributed to the fact that in the country, social ties and relationship are important to people. Therefore, the aspects to consider include the general opinions of people regarding MOOCs, the views and advice of the high-profile people, and the opinions of the important peoples. As well, another influencing factor is how the user feel motivated of enjoy using MOOCs as compared to other methods of learning. This cultivates in the fact that MOOCs platforms needs to be structured in a way that learners can enjoy the process of education and learning just like a classroom environment with the same feeling. The last aspect addressed in this research was comparing the three countries using the multi-group SEM analysis. The results revealed that key factors influencing behavioral intention to Use MOOCs were different in Poland, Thailand, and Pakistan (hypothesis 12 rejected). This implied that overall, the aspects concerning behavioral intention to use MOOCs in the three countries were different and should be treated as such. In other words, the behavioral intention to use MOOCs is influenced by different factors, and in different ways in the three countries considered–Poland, Thailand and Pakistan. Therefore, the MOOCs advocates the conditions of one country to another. It is advisable to consider the conditions of the country concerned independently. However, it was not enough to just mention that the three countries were different. It was important to highlight the specific factor under this study, on which the three countries varied, as far as the behavioral intention to adopt MOOCs in concerned. As a results, on further investigation of the individual factors, the research revealed that the factors that were different among the three countries included performance expectancy, social distancing, price value, facilitating conditions, and social influence. Majorly, these are the factors that should be treated differently among the three countries.

## 5. Conclusions

This research investigated the variables that influence behavioral intention to use MOOCs in Poland, Thailand, and Pakistan, and a comparison of the findings among these countries. The study was considered significant considering the COVID-19 pandemic, which prompted most of the educational activities in the study areas as well as the whole world conducted online. As MOOCs were an important education trend, it was critical to investigate the key aspects worth considering. The study adopted the UTAUT2 model and previous research to develop the conceptual framework, with an addition of other variables namely culture, social distancing, and absorptive capacity.

The research data was collected from the respondents in the three countries and analyzed statistically. The analysis was conducted using CFA, SEM, and multi-group SEM to compare the findings for the three countries. The model evaluation indicated that the proposed framework was acceptable and suitable for hypothesis evaluation. From the findings of the study, several achievements could be highlighted. First, the effort expectancy and culture significantly and positively influenced behavioral intention to use MOOCs. This implies that aspects such as the ease of use of technology, clarity and ability to understand by the users, flexibility to interact in the platform should be considered. As well, the three countries observe different cultural background, which influences the behavioral intention to use MOOCs. Therefore, the aspects of the rules, policies, beliefs, and norms that are observed by the concerned individuals in each country should be put into considerations. Additionally, absorptive capacity is mediated significantly by performance expectancy and effort expectancy. Facilitating conditions have a significant influence on MOOCs in both Thailand and Pakistan. This has interesting findings that the support received from the existing organization, infrastructural facility, and technical aspects are crucial in boosting the MOOCs adoption. The findings indicated that social influence has a significant influence on behavioral intention to use MOOCs in Thailand, hedonic motivation and price value have a significant influence on behavioral intention to use MOOCs in Poland, and the habit has a significant factor in Pakistan. The research indicated that the main factors influencing behavioral intention to use MOOCs were different in Poland, Thailand and Pakistan, in various factors which are performance expectancy, social distancing, price value, facilitating conditions, and social influence. Though the research is considered successful in addressing the study hypothesis, several limitations could be highlighted. The limitation was that this research was conducted in three countries of Poland, Thailand, and Pakistan, and application of the findings in other areas should be done with this consideration. Additionally, the multi-group analysis was used to answer only one hypothesis, further studies can be carried out in the future to address the use of multigroup analysis in multiple hypotheses to provide a clearer view of the MOOCs landscape in the three countries. Also, collecting a big sample size was not possible because of the complexity of collecting data in three countries. Another limitation is that the study was not carried among students who studied a certain subject; therefore, the results obtained are generalized for various subjects taught in the universities included in the sample.

The results of this research are critically important to developing the future implication in terms of practical and theoretical recommendations. From the practical perspective, this research recommends that two major factors should be considered by all countries as significant factors that influence behavioral intention to use MOOCs. These factors are effort expectancy and culture. Another practical implication considered for this research is that for the three countries, the main factors influencing their behavioral intention to use MOOCs varies when results in the three countries are juxtaposed. This implies that it is important to evaluate the situation and prevailing conditions of the concerned country separately, before implementing the MOOCs and the associated online learning practices. Specific factors that are worth evaluating differently for each of the countries include performance expectancy, social distancing, price value, facilitating conditions, and social influence. From the theoretical implications, this research adopted a modified UTAUT2 model but included three variables of culture, absorptive capacity, and social distancing. Therefore, future research should consider adopting a full UTAUT2 model and different additional variables.

## Supporting information

S1 Data (XLSX)
